# Current Understanding of Sarcopenia and Malnutrition in Geriatric Rehabilitation

**DOI:** 10.3390/nu15061426

**Published:** 2023-03-16

**Authors:** Shinta Nishioka

**Affiliations:** 1 Department of Clinical Nutrition and Food Management, Institute of Biomedical Sciences, Tokushima University Graduate School, Tokushima 770-8503, Japan; shintacks@yahoo.co.jp; Tel.: +81-88-633-7095; 2 Department of Clinical Nutrition and Food Services, Nagasaki Rehabilitation Hospital, Tokushima 770-8503, Japan

Malnutrition and sarcopenia are different but common conditions in older adults that share some underlying causes, including age-related physiological changes, decreased food intake, acute or chronic inflammation [1,2]. Both conditions are particularly prevalent in geriatric rehabilitation patients. Approximately 13% of these patients are malnourished and 47% are at risk of malnutrition [3], whereas 48% of stroke rehabilitation patients manifest sarcopenia [4]. Moreover, malnutrition and sarcopenia generally worsen outcomes of geriatric rehabilitation patients. Undernutrition can aggravate functional recovery, discharge outcomes, and swallowing function of older patients in convalescent rehabilitation wards [5,6,7,8]. Conversely, physical disabilities may degrade nutritional status, often leading to a vicious cycle of malnutrition and physical disabilities, the so-called “malnutrition-disability cycle” [9]. Similarly, sarcopenia can inhibit the functional recovery of geriatric rehabilitation patients [4,10,11].

Based on these findings, it is now hypothesized that the co-occurrence of malnutrition and sarcopenia may further worsen the prognosis. In 2012, Vandewoude et al. proposed the concept of the malnutrition–disability cycle, “the clinical presentation of both malnutrition and accelerated age-associated loss of lean body mass, strength, and/or functionality” [12]. Subsequently, several studies have confirmed the adverse effects of co-occurring malnutrition and sarcopenia in hospitalized patients [13,14]. However, the effects of simultaneous malnutrition and sarcopenia in geriatric populations and how best to diagnose and treat them are still being discussed.

This Special Issue focuses on (1) the prevalence and factors associated with malnutrition and sarcopenia in geriatric rehabilitation settings; (2) the consequences of both conditions concerning functional, nutritional, and health-related outcomes; and (3) potentially effective treatment for older rehabilitative patients with undernutrition and sarcopenia. Although this topic mainly focuses on geriatric rehabilitation, studies in other settings, e.g., acute care, long-term care, or community, are also included.

The current Special Issue includes eight articles in various fields of clinical nutrition. Yanagi et al. investigated the effect of sarcopenia on one-year mortality in critically ill older patients [15]. They show that patients with low muscle thickness (MT) and low muscle strength (MS) had worse one-year survival than patients with high MT and high MS (*p* < 0.001). In addition, combined low MT and low MS more accurately predicted one-year mortality than low MT alone. Shimizu and colleagues examined the accuracy of the Simplified Nutritional Appetite Questionnaire (SNAQ) and SNAQ for Japanese Elderly (SNAQ-JE) for malnutrition and sarcopenia screening of geriatric rehabilitation patients [16]. This cross-sectional study showed poor sensitivity (30–33%) and moderate specificity (83–86%) of the SNAQ as well as moderate sensitivity (70–73%) and poor specificity (51–54%) of the SNAQ-JE to identify malnutrition and sarcopenia. A cross-sectional study conducted by Wang et al. revealed that GLIM-defined malnutrition, using muscle mass reduction (MMR), identified by one of eight operational definitions, showed almost the same sensitivity (50.2–60.4%) and exactly the same specificity (97.9%) for malnutrition as defined by the patient-generated subjective global assessment (PG-SGA) [17]. We conducted a cross-sectional study to explore the prevalence and factors associated with co-occurring malnutrition and sarcopenia (Co-MS) in geriatric rehabilitation patients [18]. We found a very high prevalence of sarcopenia (62%), a moderate prevalence of malnutrition (29.0%), and Co-MS (23.5%). Multivariable analysis showed that onset–admission interval (odds ratio (OR): 1.04, 95% confidence interval (CI): 1.02–1.06), hospital-associated de-conditioning (OR: 4.62. 95%CI: 1.13–18.8), and Food Intake LEVEL Scale (OR: 0.83, 95%CI: 0.73–0.93) were independently associated with Co-MS. Kimura et al. examined the association between the frequency of high-protein food intake and physical performance in older adults in a cross-sectional study [19]. They found that a high food frequency score (FFS) and a high protein food frequency score (PFFS) were significantly associated with physical performance. A prospective cohort study by Paillaud examined the association of serum leptin level and nutritional status with healthcare-associated infections (HAI) in hospitalized older patients [20]. Multivariable analysis showed that serum leptin level was independently associated with HAI in women but not in men. However, the association weakened when the statistical model included serum albumin or malnutrition. A systematic review and meta-analysis of the prevalence of undernutrition, frailty, and sarcopenia in community-dwelling older adults was conducted by Almohaisen et al. [21]. Their literature search found 37 studies and showed that 17%, 13%, and 14% of participants experienced malnutrition, frailty, and sarcopenia, respectively. Finally, Irisawa and Mizushima conducted a prospective cohort study of the association between nutritional status, body composition, muscle strength, and functional recovery in patients after proximal femoral fracture [22]. Multivariable analysis showed that a higher Geriatric Nutritional Risk Index and phage angle were associated with higher Functional Independence Measure (FIM) scores.

In summary, this Special Issue provides evidence for the adverse effects of malnutrition, sarcopenia, and their co-occurrence, as well as assessment methods for these conditions in older adults with or without rehabilitation. Further investigation is warranted to restore nutritional status, muscle mass, and function for older adults through a combination of rehabilitation and nutrition therapy [23].
